# High-Throughput Sequencing Reveals Circulating miRNAs as Potential Biomarkers of Kidney Damage in Patients with Systemic Lupus Erythematosus

**DOI:** 10.1371/journal.pone.0166202

**Published:** 2016-11-11

**Authors:** Elkin Navarro-Quiroz, Lisandro Pacheco-Lugo, Hernan Lorenzi, Yirys Díaz-Olmos, Lisneth Almendrales, Edwin Rico, Roberto Navarro, Pierine España-Puccini, Antonio Iglesias, Eduardo Egea, Gustavo Aroca

**Affiliations:** 1 Grupo de Nefrología, Universidad Simón Bolívar, Barranquilla, Colombia; 2 Infectious Diseases Department, J. Craig Venter Institute, Rockville, Maryland, United States of America; 3 Grupo de Genética, Universidad Cooperativa de Colombia, Santa Marta, Colombia; 4 Monómeros, Barranquilla, Colombia; 5 Unidad de Reumatología, Universidad Nacional de Colombia, Bogotá, Colombia; 6 Grupo de Investigación en Inmunología y Biología Molecular, Universidad del Norte, Barranquilla, Colombia; 7 Clínica de la Costa, Barranquilla, Colombia; Peking University First Hospital, CHINA

## Abstract

Renal involvement is one of the most severe manifestations of systemic lupus erythematosus (SLE). Renal biopsy is the gold standard when it comes to knowing whether a patient has lupus nephritis, and the degree of renal disease present. However, the biopsy has various complications, bleeding being the most common. Therefore, the development of alternative, non-invasive diagnostic tests for kidney disease in patients with SLE is a priority. Micro RNAs (miRNAs) are differentially expressed in various tissues, and changes in their expression have been associated with several pathological processes. The aim of this study was to identify changes in the abundance of miRNAs in plasma samples from patients with lupus nephritis that could potentially allow the diagnosis of renal damage in SLE patients. This is an observational case-control cross-sectional study, in which we characterized the differential abundance profiles of miRNAs among patients with different degrees of lupus compared with SLE patients without renal involvement and healthy control individuals. We found 89 miRNAs with changes in their abundance between lupus nephritis patients and healthy controls, and 17 miRNAs that showed significant variations between SLE patients with or without renal involvement. Validation for qPCR of a group of miRNAs on additional samples from lupus patients with or without nephritis, and from healthy individuals, showed that five miRNAs presented an average detection sensitivity of 97%, a specificity of 70.3%, a positive predictive value of 82.5%, a negative predictive value of 96% and a diagnosis efficiency of 87.9%. These results strongly suggest that miR-221-5p, miR-380-3p, miR-556-5p, miR-758-3p and miR-3074-3p are potential diagnostic biomarkers of lupus nephritis in patients with SLE. The observed differential pattern of miRNA abundance may have functional implications in the pathophysiology of SLE renal damage.

## Introduction

Lupus nephritis (LN) is the commonest and one of the most serious manifestations of SLE [1,2], with reports of 5-year renal survivals ranging from 46% to 95% with treatment [3]. Renal involvement in SLE remains a significant cause of morbidity and mortality. The prognosis of LN is particularly bad in certain ethnic groups, such as African-Americans and Hispanics [4].

The pathogenesis of LN is a complex process, involving deposition of autoantibodies in the glomerulus, activation of complement and macrophages, cell proliferation, and production of extracellular matrix proteins, pro-inflammatory cytokines, and chemokines, which are then linked through multiple mechanisms to cause tubular damage, tubulointerstitial inflammation, and fibrosis. [5]

Renal biopsy is the gold standard for providing information on the histological classes of LN and the relative degree of activity and chronicity in the glomeruli. However, it is invasive and serial biopsies are impractical in the monitoring of LN. Thus, novel biomarkers that are able to discriminate lupus renal activity and its severity, predict renal flares, and monitor treatment response and disease progress are clearly necessary. Micro RNAs are short non-coding RNA sequences that regulate gene expression by blocking protein translation or inducing mRNA degradation [6]. The variation of miRNA levels could cause the dysregulation of a broad range of targeted genes that may lead to disease. The altered expression of miRNAs in kidney during pathological processes makes miRNAs a valuable new tool for understanding, diagnosing, and discovering alternative therapies for SLE and LN. Several studies have reported the potential of miRNAs as biomarkers of renal injury in SLE [7–9]. In this study, we evaluated the miRNAs circulating in plasma samples of individuals with different stages of renal involvement using a high throughput sequencing approach, and we found a group of miRNAs whose expression pattern correlates with renal involvement in patients with SLE. To our knowledge, this is the first study that has used a group of patients with different involvement of renal injury in SLE to compare circulating miRNAs using large-scale sequencing.

## Materials and Methods

### Sample

The present study was based on a cohort of Colombian patients with lupus nephritis (www.nefrored.org). Renal histopathology was classified according to the 2003 revised criteria for glomerulonephritis of SLE, which was published by International Society of Nephrology/Renal Pathology Society [10]. Written informed consent was obtained prior to enrolment from all adult individuals participating in the study. The study protocol was reviewed and approved by the ethics review board at Simon Bolivar University.

### Sample processing

Whole blood (10 mL) from subjects was collected via a direct venous puncture into tubes with ethylenediaminetetraacetic acid (EDTA) as an anticoagulant. Blood was processed for the isolation of plasma within 4 h of collection. The blood was processed by spinning at 2,000 x g for 10 min at room temperature. Then, the plasma was transferred to a RNase-free tube and stored at –80°C.

### RNA isolation from plasma

The plasma was thawed on ice. Total RNA was isolated from 600 μl of plasma using miRVana PARIS Kit (Ambion), following the manufacturer instructions. RNA concentration was measured by Nanodrop 2000 spectrophotometer (Thermo Scientific) and stored at −80°C. All materials and solutions were handled in RNase-free conditions. All solutions were prepared in RNase-free water and all methods were carried out in accordance with the approved guidelines.

### miRNA sequencing and differential gene expression analysis

Illumina barcoded miRNA sequencing libraries were prepared from extracted miRNA samples using TruSeq Small RNA Library Prep Kit (Illumina^®^) following manufacturer instructions. Sequencing libraries were sequenced in two pools of 15 samples each per sequencing runs with a NextSeq apparatus to generate ~16 million single-end 75 bp reads per sample. Afterwards, sequencing reads were processed with CLC Genomics Workbench software (https://www.qiagenbioinformatics.com/products/clc-genomics-workbench/) to obtain the final counts of miRNAs present in teach sample. Briefly, adapter sequences were removed from sequencing reads and the remaining sequences were compared against the miRBase database (www.miRBase.org) and the Ensembl human non-coding RNA annotation (version GRCh37.75) with CLC for miRNA gene identification, annotation and quantification. Differential gene expression analysis between groups of interest was carried out with the R package EdgeR.[11]

### qPCR validation of plasma miRNA as biomarkers of renal involvement in SLE

To validate the sequencing results, we assessed the miRNAs with differential abundance using qRT-PCR in 180 samples (patients with SLE, n = 40; LN, n = 100 and healthy controls, n = 40). cDNA was synthesized from total RNA using the "TaqMan" MicroRNA Reverse Transcription Kit (Applied Biosystems, Foster City, CA). The reaction conditions and the amounts of RNA, primers, dNTPs, buffer and other constituents of the reverse transcription reaction were standardized in our laboratory. For each 15-μL RT-reaction, we combined the RT mastermix with total RNA using a the ratio of 7 μL RT master mix: 5 μL total RNA (1 to 10 ng per reaction) and added 3 μL of 5✕ RT primer from each assay set into the corresponding RT reaction. All the reactions were incubated in a 96-well plate at 95°C for 10 min, followed by 40 cycles of 95°C for 15 s and 60°C for 15 s. Quantitation of miRNAs expression was performed by the method of double delta Ct. To normalize qPCR copy-number data we used hsa-miR-26b-5p (478418_mir, Cat. # A25576, Thermo Fisher Scientific). The results are expressed in terms of sensitivity (number of true positive test results in all patients with LN), specificity (number of true negative test results in all patients without LN), positive predictive value (number of true positive test results of all positive test results observed), negative predictive value (number of true negative test results of all negative test results observed), and efficiency (number of true positive and negative test results of all positive and negative test results observed). Receiver operating characteristics (ROC) curves were obtained by plotting sensitivity against 1-specificity. The diagnostic values of five candidate miRNAs were evaluated by area under the ROC curve (AUC).

### Functional analysis

miRNAs miR-221-5p, miR-380-3p, miR-556-5p, miR-758-3p and miR-3074-3p were functionally analyzed through “pathways Union”, using the DIANA-miRPath v3 program. [12]

## Results

### General data of the subjects

A total of 180 plasma samples, including 20 patients LN class II (LNII), 40 LN class III (LNIII), 40 LN class IV (LNIV) (Table 1) and 40 SLE non-LN (LNN) (Table 2) as well as 40 healthy individuals (CTL), were included in this study. LN activity was evaluated based on the Systemic Lupus Erithematous Disease Activity Index (SLEDAI) [13]. Patients SLE with recently diagnosed renal disease used steroids infusion, with the transition to the oral steroids at reduce doses, or were administered only oral steroids. Furthermore, the immunosuppressive therapy contained cyclophosphamide, azathioprine, cyclosporin A, mycophenolate mofetil, and chloroquine. In order to keep remission, patients utilized oral steroids or oral steroids mixed with mycophenolate mofetil or azathioprine [12]. 10 plasma samples from LNN, 7 plasma samples from CTL, 4 plasma samples from LNII, 4 plasma samples from LNIII, and 6 plasma samples from LNIV were used to high-throughput sequencing.

**Table 1:** Distribution of cases falling in each class in ISN/RPS classification (2003).

Class		Brief description	Number	Age (median)	Gender (F)
II (mesangial proliferative LN)		Purely mesangial hypercellularity of any degree OR mesangial matrix expansion by light microscopy with mesangial immune deposits	20	24	19
III (focal LN)	III (A)	Active lesions	0	0	0
	III (A/C)	Active and chronic lesions	12	32	11
	III (C)	Chronic inactive lesions	28	34	26
IV (diffuse LN)	IV-S (A)	Active: diffuse segmental proliferative LN	2	27	2
	IV-G (A)	Active: diffuse global proliferative LN	8	24	8
	IV-S (A/C)	Active and chronic: diffuse segmental proliferative and sclerosing LN	4	22	4
	IV-G (A/C)	Active and chronic: diffuse global proliferative and sclerosing LN	16	27	15
	IV-S (C)	Chronic inactive: diffuse segmental sclerosing LN	1	16	1
	IV-G (C)	Chronic inactive: diffuse global sclerosing LN	9	28	9

**Table 2:** Characteristics of the study LNN group in terms of age, gender, and disease activity measured by SLEDAI scale.

	SLEDAI
SLEDAI score	>5
Group size	40
Mean SLEDAI	14
Median SLEDAI	13
Min–Max SLEDAI	7–29
Mean age	36.7 ± 14.9
Median age	32
Gender	♀: 40 (100%)
	♂: 0 (0%)

### Differential abundance patterns of miRNAs in SLE individuals

**Fig 1:**
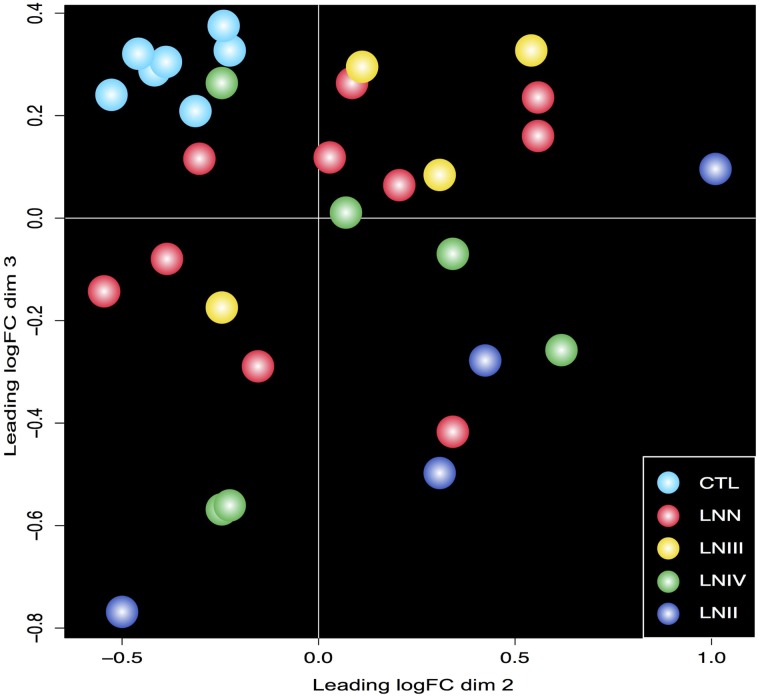
Principal Component Analysis (PCA) of study groups miRNA profiles. Two-dimensional PCA was used to determine whether control individuals (CTL) could be distinguished from study subjects.

Our analysis used next generation sequencing to comprehensively examine the plasma abundance of miRNAs in patients with LNII, LNIII or LNIV compared with plasma miRNA levels in lupus patients with no nephritis (LNN) or control healthy individuals (CTL). Principle Component Analysis (PCA) of miRNA profiles showed that samples from LN and LNN patients separate from healthy control individuals (Fig 1).

**Fig 2:**
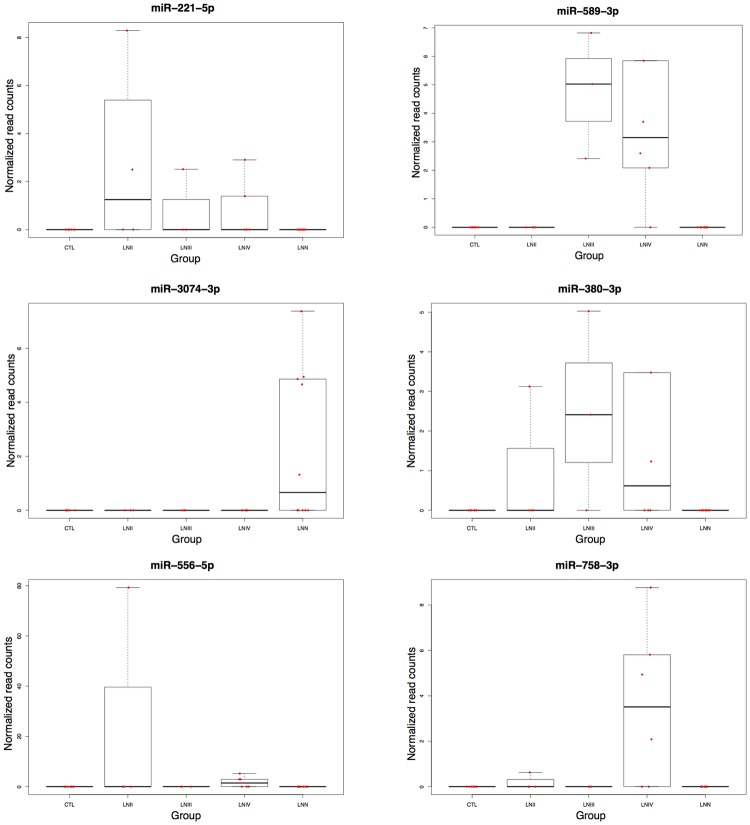
Box plots depict normalized reads counts of candidate miRNAs between different groups of samples.

We found 89 miRNAs whose abundance was significantly different in patients with LN when compared with CTL individuals, and another 17 miRNAs differently abundant in patients with LN when compared with the LNN group (S1 Table, supplementary data). Volcano plots were used to represent the data distribution between study subjects and CTL individuals (S1 Fig), and intra study groups (S2 Fig). We evaluated the normalized expression for each miRNA across the 5 groups of samples (data not shown) and we selected miR- miR-221-5p, miR-380-3p, miR-556-5p, miR-758-3p because they presented reads counts in LN patients but not in LNN and CTL individuals, and the miR-3074-3p with reads counts in LNN but not in LN and CTL group, and we used these miRNAs to validate the diagnosis made by renal biopsy (Fig 2).

### Validation of miRNAs as biomarkers of kidney damage associated with lupus erythematosus

**Fig 3:**
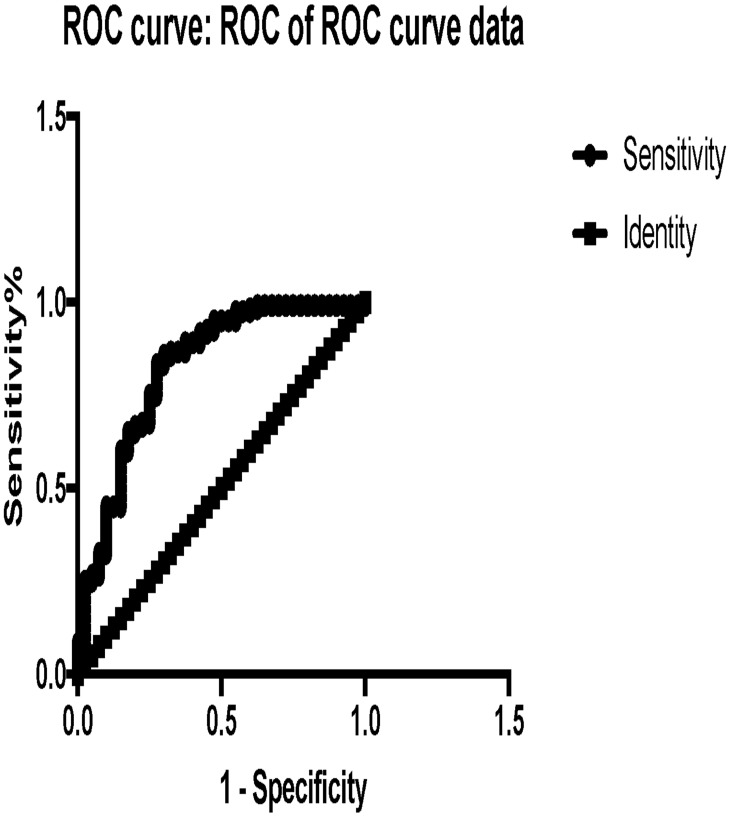
Receiver operating characteristic (ROC) curves to assess the utility of miRNAs to differentiate LN from LNN patients.

Based on their expression profiles, five miRNAs that were present in plasma from LN patients but not in LNN or CTL individuals were selected as potential biomarkers of kidney damage (miR-221-5p, miR-380-3p, miR-556-5p, miR-758-3p and miR-3074-3p) and further validated by RT-qPCR in 100 plasma samples from LN patients diagnosed by renal biopsy (gold standard) (Table 1). These patients were classified according to the criteria of International Society of Nephrology/Renal Pathology Society (ISN/RPS) 2003 [14]. The same 5 miRNAs were further corroborated by RT-qPCR in 40 plasma samples from LNN patients and 40 plasma samples from healthy individuals. These five miRNAs as a group were able to discriminate between LN and LNN/CTL samples with very good sensitivity (97%), specificity (70.3%), positive predictive value (82.5%), negative predictive value (96%) and diagnostic efficiency (87.9%). The area under the ROC curve (AUC) is a measure of discrimination; a model with a high area under the ROC curve suggests that the model is able to accurately predict the value of an observation's response. When we compared the combined performance of miR-221-5p, miR-380-3p, miR-556-5p, miR-758-3p and miR-3074-3p we got an AUC of 0.82 (95% CI: 0.7–0.9; p-value <0.0001) that according to Hosmer and Lemeshow [15] it allows excellent discrimination of patients with LN of the patients with LNN (Fig 3). Even though these findings do not allow us to determine the class of lupus nephritis, which was our initial aim, they support the miRNAs miR-221-5p, miR-380-3p, miR-556-5p, miR-758-3p and miR-3074-3p as potential biomarkers of kidney damage in patients with systemic lupus erythematosus.

### Functional analysis

To gain insights into the biological processes modulated by the 5 candidate biomarker miRNAs, we perform a gene enrichment analysis with the program DIANA-miRPath v3 [12]. Only miR-221-5p and miR-556-5p presented significant hits, with prion-diseases pathway being the most significant (p-value = 8.32e-30), and where the expression of five genes involved in this pathway are predicted to be regulated by hsa-miR-221-5p.

## Discussion

Current laboratory markers for lupus nephritis such as proteinuria, urine protein-to-creatinine ratio, creatinine clearance, anti-dsDNA, and complement levels are unsatisfactory [16] and they lack sensitivity and specificity for differentiating renal activity and damage in lupus nephritis [5]. Lupus nephritis is one of the most severe manifestations of systemic lupus erythematosus (SLE), which is associated with significant morbidity and mortality in SLE patients. The development of diagnostic tests with high sensitivity that enables the early detection of kidney injury in SLE patients instead of renal biopsy remain as a critical issue. In this study we identified a collection of five miRNAs (miR-221-5p, miR-380-3p, miR-556-5p, miR-758-3p and miR-3074-3p) with differential expression in a cohort of Colombians patients with SLE. Our results support the promising potential of this miRNAs as biomarkers of LN and their usefulness for translation into clinical practice.

In recent years miRNAs have been shown to be involved in a wide range of biological process, including several human diseases [17]. A probable link between miRNAs and SLE have been suggested. Dai, et al. [18] and Te, et al. [19] using microarray analysis reported the presence of miRNAs differentially expressed in SLE and LN individuals, respectively.

In this study, using high-throughput sequencing we isolated and analyzed miRNA in plasma samples obtained from SLE-affected patients with different grades of renal involvement (LN II, III and IV) as well as unaffected controls. Our data indicated that hsa-miR-221-5p, hsa-miR-380-3p, hsa-miR-556-5p, hsa-miR-758-3p and hsa-miR-3074-3p showed remarkably high diagnostic accuracy for differentiating LN cases from LNN and therefore, they are potential biomarkers of kidney disease in patients with SLE. The data described in this work need to be validated using larger populations. However, some miRNAs with significant differential abundance has been previously associated with LN [19–20].

In an effort to explain the physiological significance of the five miRNAs found to be differentially enriched in plasma in our study we mined the miRBase miRNA database (http://www.mirbase.org/) in order to identify validated targets of the five candidate miRNAs biomarkers (Table 3).

**Table 3:** Potential gene targets for the five candidate miRNA biomarkers.

The potential gene target for the primary differentially expressed miRNA.	
Major miRNA identified	Potential molecular Targets
hsa-miR-221-5p	BCL2L2, NUFIP2, GRIK2, TMEM115, KIF3B
hsa-miR-380-3p	HECA, RBM26, SLC6A15, EIF4E, CRISPLD1
hsa-miR-556-5p	LGALSL, CSRNP2, HIPK2
hsa-miR-758-3p	PALM2-AKAP2, HIPK3, HNRNPU
hsa-miR-3074-3p	SMIM13, BTBD1

>0.7 prediction score (www.tarbase.org)

LGALSL (lectin, galactoside-binding-like) was identified as a target of hsa-miR-556-5p. While the classical complement pathway is the foundation of pathogenesis of lupus nephritis, the alternative pathway and lectin pathway appear to play a role in the progression of glomerular damage [21]. Patients with glomerular deposition of properdin, a positive regulator of the alternative pathway, and patients with mannose binding lectin/L-ficolin show increased urinary protein excretion [21]. Also, we found that the predicted target of hsa-miR-758-3p is the transcription factor E2F1 and in the case of the miRNA hsa-miR-423-5p are the nucleic acid binding proteins Hox-B8 and Hox-A7. E2F transcription factors are important regulators of proliferation, differentiation and apoptosis. E2F transcription factors and homeobox proteins were previously identified as targets of miRNA profiling in the work published by Dai et al [14] The relationship between transcription factors and SLE was approached by Azkargorta et al.[22], who found a similarity between the metabolic state for E2F2 deficient T-lymphocytes and that of lupus T-cells, which added new insights into the relationship between SLE and E2F2 deficiency.

Notably, miRNAs modulate the expression of targeted genes to an optimum level, rather than participating in on/off decisions in the inflammatory response [23], providing unique roles in rheumatic diseases by regulating inflammation. Systemic lupus erythematosus is a chronic autoimmune inflammatory disease characterized by a myriad of immunoregulatory abnormalities that lead to injuries of tissues and organs. To date, the etiology of lupus nephritis is unknown, and the characterization of differential expression profiles of miRNAs will contribute to the clarification of the physiological mechanisms involved in the development of lupus and of renal diseases associated with it. Here we show five pathways in which one or more genes are predicted to be regulated by at least one of the 5 miRNAs validated for the diagnosis of renal involvement in patients with SLE. For example, the interactions of hsa-miR-221-5p with genes PRNP, MAPK3, LAMC1, MAP2K1, and IL1A, have been validated experimentally [24–26]. The observed increase in the abundance of miR-221-5p may be a homeostatic mechanism to counteract an increase in the expression of laminin-1, which has been described to replace laminin-11 in glomerular basement membrane as a result of overproduction of TGFB1 in patients with lupus nephritis [27,28]. This makes nucleosomes readily bind to laminin-1 through their β1 chain and the trapped nucleosomes can then be bound by autoantibodies that increase T-cell-dependent autoimmune responses, sharpening the early pathogenesis of lupus nephritis. Additionally, the interaction between hsa-miR-221-5p and IL1A transcripts leads to diminished levels of IL1A, serum Ca2+, serum IgG1, IgE, IL17, and IL4. Since IL1A participates in the modulation of extracellular calcium homeostasis fluid, as described direct relation between IL1A concentrations and Ca2+ [29] also markedly induced robust and durable primary and secondary CD4 T cell response, with an increase in cells producing IL17 and IL4, as well as serum IgG1 and IgE by B cells [30]. This may be associated with a decrease in apoptosis, previously described by other authors in patients with lupus nephritis [31].

The search for non-invasive markers for diagnosis of SLE is currently a priority. In this study we have demonstrated that the combined use of the miRNAs miR-221-5p, miR-380-3p, miR-556-5p, miR-758-3p and miR-3074-3p can serve as noninvasive biomarkers for the diagnosis of renal involvement in SLE. Our strategy of using highthroughput sequencing (Illumina) followed by qRT-PCR validation shown to be a successful approach to identifying plasma miRNA profiles as biomarkers for the diagnosis of lupus nephritis.

## Supporting Information

S1 Fig Volcano plots representing differential expression analysis of miRNA between patient groups and control individuals. **A)** Lupus Non Nephritis (LNN) patients compared to healthy control individuals (CTL). **B)** Lupus Nephritis II (LNII) patients compared to CTL individuals. Orange dots, miRNAs with log2 fold change > 1; red dots, miRNAs with q-value < 0.2; green dots, miRNAs with q-value < 0.05 and Log2 fold change > 1. The graphics show the presence of miRNAs (represented by dots) that have a differential abundance profile statistically significant as evidenced by the p-values and the fold change values (miRNAs of interest are highlighted in green color).
(EPS)

S2 Fig Volcano plots representing differential expression analysis of miRNA between patient groups and control individuals. **A)** Lupus Nephritis III (LNIII) patients compared to CTL individuals. **B)** Lupus Nephritis IV (LNIV) patients compared to CTL individuals. Orange dots, miRNAs with log2 fold change > 1; red dots, miRNAs with q-value < 0.2; green dots, miRNAs with q-value < 0.05 and Log2 fold change > 1. The graphics show the presence of miRNAs (represented by dots) that have a differential abundance profile statistically significant as evidenced by the p-values and the fold change values (miRNAs of interest are highlighted in green color).
(EPS)

S3 Fig Volcano plots representing differential expression analysis of miRNA between patient group with SLE. **A)** LNN patients compared to LNII. **B)** LNN patients compared to LNIII. Orange dots, miRNAs with log2 fold change > 1; red dots, miRNAs with q-value < 0.2; green dots, miRNAs with q-value < 0.05 and Log2 fold change > 1. The graphics show the presence of miRNAs (represented by dots) that have a differential abundance profile statistically significant as evidenced by the p-values and the fold change values (miRNAs of interest are highlighted in green color).
(EPS)

S4 Fig Volcano plots representing differential expression analysis of miRNA between patient group with SLE. **A)** LNN patients compared to LNIV. Orange dots, miRNAs with log2 fold change > 1; red dots, miRNAs with q-value < 0.2; green dots, miRNAs with q-value < 0.05 and Log2 fold change > 1. The graphics show the presence of miRNAs (represented by dots) that have a differential abundance.
(EPS)

S1 Table Circulating miRNAs whose abundance was significantly different between study groups. (DOCX)

## References

[1] Mok CC, Tang SSK. Incidence and predictors of renal disease in Chinese patients with systemic lupus erythematosus. Am J Med. 2004;117: 791–5. 10.1016/j.amjmed.2004.04.029 15541328

[2] Mok CC, Tang SSK, To CH, Petri M. Incidence and risk factors of thromboembolism in systemic lupus erythematosus: a comparison of three ethnic groups. Arthritis Rheum. 2005;52: 2774–82. 10.1002/art.21224 16142761

[3] Korbet SM, Lewis EJ, Schwartz MM, Reichlin M, Evans J, Rohde RD. Factors predictive of outcome in severe lupus nephritis. Lupus Nephritis Collaborative Study Group. Am J Kidney Dis. 2000;35: 904–14. Available: http://www.ncbi.nlm.nih.gov/pubmed/10793026 10793026 10.1016/s0272-6386(00)70262-9

[4] Dooley MA, Hogan S, Jennette C, Falk R. Cyclophosphamide therapy for lupus nephritis: Poor renal survival in black Americans. Kidney Int. 1997;51: 1188–1195. 10.1038/ki.1997.162 9083285

[5] Li Y, Fang X, Li Q-Z. Biomarker profiling for lupus nephritis. Genomics Proteomics Bioinformatics. 2013;11: 158–65. 10.1016/j.gpb.2013.05.003 23732627 PMC4357827

[6] Meister G, Tuschl T. Mechanisms of gene silencing by double-stranded RNA. Nature. 2004;431: 343–9. 10.1038/nature02873 15372041

[7] Dai Y, Sui W, Lan H, Yan Q, Huang H, Huang Y. Comprehensive analysis of microRNA expression patterns in renal biopsies of lupus nephritis patients. Rheumatol Int. 2009;29: 749–54. 10.1007/s00296-008-0758-6 18998140

[8] Lu J, Kwan BC-H, Lai FM-M, Tam L-S, Li EK-M, Chow K-M, et al Glomerular and tubulointerstitial miR-638, miR-198 and miR-146a expression in lupus nephritis. Nephrology (Carlton). 2012;17: 346–51. 10.1111/j.1440-1797.2012.01573.x 22295894

[9] Te JL, Dozmorov IM, Guthridge JM, Nguyen KL, Cavett JW, Kelly JA, et al Identification of unique microRNA signature associated with lupus nephritis. PLoS One. Public Library of Science; 2010;5: e10344 10.1371/journal.pone.0010344 20485490 PMC2867940

[10] Weening JJ. The Classification of Glomerulonephritis in Systemic Lupus Erythematosus Revisited. J Am Soc Nephrol. 2004;15: 241–250. 10.1097/01.ASN.0000108969.21691.5D 14747370

[11] Robinson MD, McCarthy DJ, Smyth GK. edgeR: a Bioconductor package for differential expression analysis of digital gene expression data. Bioinformatics. 2010;26: 139–40. 10.1093/bioinformatics/btp616 19910308 PMC2796818

[12] Vlachos IS, Zagganas K, Paraskevopoulou MD, Georgakilas G, Karagkouni D, Vergoulis T, et al DIANA-miRPath v3.0: deciphering microRNA function with experimental support. Nucleic Acids Res. 2015;43: W460–6. 10.1093/nar/gkv403 25977294 PMC4489228

[13] Gladman DD, Ibañez D, Urowitz MB. Systemic lupus erythematosus disease activity index 2000. J Rheumatol. 2002;29: 288–91. Available: http://www.ncbi.nlm.nih.gov/pubmed/11838846 11838846

[14] Weening JJ, D’Agati VD, Schwartz MM, Seshan S V., Alpers CE, Appel GB, et al The Classification of Glomerulonephritis in Systemic Lupus Erythematosus Revisited. J Am Soc Nephrol. American Society of Nephrology; 2004;15: 241–250. 10.1097/01.ASN.0000108969.21691.5D 14747370

[15] Hosmer DW, Lemeshow S, Sturdivant RX. Applied logistic regression.

[16] Mok CC, Mok CC. Biomarkers for lupus nephritis: a critical appraisal. J Biomed Biotechnol. Hindawi Publishing Corporation; 2010;2010: 638413 10.1155/2010/638413 20414362 PMC2857808

[17] Ardekani AM, Naeini MM. The Role of MicroRNAs in Human Diseases. Avicenna J Med Biotechnol. Avicenna Research Institute; 2010;2: 161–79. Available: http://www.ncbi.nlm.nih.gov/pubmed/23407304 PMC3558168 23407304

[18] Dai Y, Huang Y-S, Tang M, Lv T-Y, Hu C-X, Tan Y-H, et al Microarray analysis of microRNA expression in peripheral blood cells of systemic lupus erythematosus patients. Lupus. 2007;16: 939–46. 10.1177/0961203307084158 18042587

[19] Te JL, Dozmorov IM, Guthridge JM, Nguyen KL, Cavett JW, Kelly JA, et al Identification of unique microRNA signature associated with lupus nephritis. PLoS One. 2010;5: e10344 10.1371/journal.pone.0010344 20485490 PMC2867940

[20] Dai Y, Sui W, Lan H, Yan Q, Huang H, Huang Y. Comprehensive analysis of microRNA expression patterns in renal biopsies of lupus nephritis patients. Rheumatol Int. 2009;29: 749–54. 10.1007/s00296-008-0758-6 18998140

[21] Sato N, Ohsawa I, Nagamachi S, Ishii M, Kusaba G, Inoshita H, et al Significance of glomerular activation of the alternative pathway and lectin pathway in lupus nephritis. Lupus. 2011;20: 1378–86. 10.1177/0961203311415561 21893562

[22] Azkargorta M, Arizmendi JM, Elortza F, Alkorta N, Zubiaga AM, Fullaondo A. Differential proteome profiles in E2F2-deficient T lymphocytes. Proteomics. 2006;6 Suppl 1: S42–50. 10.1002/pmic.200500438 16544283

[23] Baltimore D, Boldin MP, O’Connell RM, Rao DS, Taganov KD. MicroRNAs: new regulators of immune cell development and function. Nat Immunol. 2008;9: 839–45. 10.1038/ni.f.209 18645592

[24] Balakrishnan I, Yang X, Brown J, Ramakrishnan A, Torok-Storb B, Kabos P, et al Genome-wide analysis of miRNA-mRNA interactions in marrow stromal cells. Stem Cells. 2014;32: 662–73. 10.1002/stem.1531 24038734 PMC4127404

[25] Karginov F V, Hannon GJ. Remodeling of Ago2-mRNA interactions upon cellular stress reflects miRNA complementarity and correlates with altered translation rates. Genes Dev. 2013;27: 1624–32. 10.1101/gad.215939.113 23824327 PMC3731550

[26] Kameswaran V, Bramswig NC, McKenna LB, Penn M, Schug J, Hand NJ, et al Epigenetic regulation of the DLK1-MEG3 microRNA cluster in human type 2 diabetic islets. Cell Metab. 2014;19: 135–45. 10.1016/j.cmet.2013.11.016 24374217 PMC3932527

[27] Jones B. Lupus nephritis: Nucleosomes trapped by aberrantly expressed laminin-β1. Nat Rev Nephrol. 2014;10: 4 10.1038/nrneph.2013.246 24247286

[28] Olin AI, Mörgelin M, Truedsson L, Sturfelt G, Bengtsson AA. Pathogenic mechanisms in lupus nephritis: Nucleosomes bind aberrant laminin β1 with high affinity and colocalize in the electron-dense deposits. Arthritis Rheumatol (Hoboken, NJ). 2014;66: 397–406. 10.1002/art.38250 24504812

[29] Sabatini M, Boyce B, Aufdemorte T, Bonewald L, Mundy GR. Infusions of recombinant human interleukins 1 alpha and 1 beta cause hypercalcemia in normal mice. Proc Natl Acad Sci U S A. 1988;85: 5235–9. Available: http://www.pubmedcentral.nih.gov/articlerender.fcgi?artid=281724&tool=pmcentrez&rendertype=abstract 3260671 10.1073/pnas.85.14.5235 PMC281724

[30] Ben-Sasson SZ, Hu-Li J, Quiel J, Cauchetaux S, Ratner M, Shapira I, et al IL-1 acts directly on CD4 T cells to enhance their antigen-driven expansion and differentiation. Proc Natl Acad Sci U S A. 2009;106: 7119–24. 10.1073/pnas.0902745106 19359475 PMC2678417

[31] Soto H, Mosquera J, Rodríguez-Iturbe B, Henriquez La Roche C, Pinto A. Apoptosis in proliferative glomerulonephritis: decreased apoptosis expression in lupus nephritis. Nephrol Dial Transplant. 1997;12: 273–80. Available: http://www.ncbi.nlm.nih.gov/pubmed/9132644 9132644 10.1093/ndt/12.2.273

