# Supplementary material for: High-Throughput Sequencing Reveals Circulating miRNAs as Potential Biomarkers of Kidney Damage in Patients with Systemic Lupus Erythematosus
Source: PLoS One. 2016 Nov 11;11(11):e0166202. doi: 10.1371/journal.pone.0166202 (PMC5106044; doi:10.1371/journal.pone.0166202)
Supplement: S1 Table — (DOCX) [file pone.0166202.s005.docx]

**S1 Table. Circulating** **miRNAs whose abundance was significantly different between study groups**

| **Groups compared** | **ID** | **Log2 Fold Changes** | **p-value** | **q-value*** |
| --- | --- | --- | --- | --- |
| **LNII_vs_LNN** | miR-144-5p | 2,8 | 0,0002 | 0,0497 |
|  | miR-144-3p | 2,6 | 0,0003 | 0,0497 |
|  | miR-550b-2-5p | 3,3 | 0,0004 | 0,0575 |
|  | miR-92a-3p | 2,1 | 0,0005 | 0,0575 |
|  | miR-219a-5p | -5,7 | 0,0023 | 0,1185 |
|  | miR-375-3p | 2,2 | 0,0029 | 0,1338 |
|  | miR-556-5p | -5,7 | 0,0053 | 0,1758 |
|  | miR-221-5p | -3,4 | 0,0058 | 0,1758 |
|  | miR-185-3p | 4,9 | 0,0058 | 0,1758 |
|  | miR-139-3p | -3,2 | 0,0060 | 0,1758 |
| **LNIV_vs_LNN** | miR-375-3p | 2,3 | 0,0002 | 0,0460 |
|  | miR-758-3p | -3,8 | 0,0002 | 0,0460 |
|  | miR-6741-3p | 4,3 | 0,0009 | 0,1011 |
|  | miR-183-5p | 2,1 | 0,0013 | 0,1157 |
|  | miR-380-3p | -3,4 | 0,0023 | 0,1680 |
|  | miR-3074-3p | 3,3 | 0,0024 | 0,1680 |
|  | miR-487b-3p | -2,9 | 0,0027 | 0,1680 |
| **LNIV_vs_CTL**  **LNIV_vs_CTL**  **LNIV_vs_CTL** | miR-183-5p | -3,4 | 0,0000 | 0,0008 |
|  | miR-145-5p | 3,2 | 0,0000 | 0,0009 |
|  | miR-584-5p | 3,0 | 0,0000 | 0,0011 |
|  | miR-6087 | 6,0 | 0,0000 | 0,0036 |
|  | miR-1260b | 3,1 | 0,0001 | 0,0076 |
|  | miR-375-3p | -2,5 | 0,0002 | 0,0115 |
|  | miR-550b-2-5p | -2,4 | 0,0002 | 0,0118 |
|  | miR-199a-5p | 2,1 | 0,0004 | 0,0186 |
|  | miR-6741-3p | -4,8 | 0,0005 | 0,0207 |
|  | miR-4511 | -4,2 | 0,0005 | 0,0207 |
|  | miR-4732-3p | -2,1 | 0,0008 | 0,0237 |
|  | miR-410-3p | 2,4 | 0,0009 | 0,0268 |
|  | miR-485-5p | 4,8 | 0,0010 | 0,0268 |
|  | miR-543 | 4,6 | 0,0011 | 0,0286 |
|  | miR-125b-5p | 2,3 | 0,0012 | 0,0296 |
|  | miR-550a-5p | -3,0 | 0,0013 | 0,0303 |
|  | miR-153-3p | 6,7 | 0,0013 | 0,0305 |
|  | miR-323b-3p | 2,9 | 0,0015 | 0,0342 |
|  | miR-106a-5p | -2,7 | 0,0017 | 0,0360 |
|  | miR-7977 | 3,1 | 0,0019 | 0,0381 |
|  | miR-369-5p | 3,2 | 0,0020 | 0,0390 |
|  | miR-589-3p | 3,9 | 0,0024 | 0,0436 |
|  | miR-3942-5p | 4,3 | 0,0026 | 0,0458 |
|  | miR-107-3p | 3,0 | 0,0029 | 0,0472 |
|  | miR-361-3p | 5,9 | 0,0031 | 0,0472 |
|  | miR-381-3p | 2,9 | 0,0041 | 0,0576 |
|  | miR-1296-5p | 4,0 | 0,0051 | 0,0680 |
|  | miR-758-3p | 3,8 | 0,0062 | 0,0755 |
|  | miR-5119 | 2,1 | 0,0062 | 0,0755 |
|  | miR-4446-3p | 2,4 | 0,0067 | 0,0783 |
|  | miR-1306-5p | -2,8 | 0,0083 | 0,0884 |
|  | miR-134-5p | 2,4 | 0,0085 | 0,0888 |
|  | miR-335-3p | 2,3 | 0,0095 | 0,0979 |
|  | miR-126b-5p | -4,1 | 0,0100 | 0,1012 |
|  | miR-433-3p | 3,5 | 0,0105 | 0,1042 |
|  | miR-1983 | 2,9 | 0,0117 | 0,1127 |
|  | miR-148b-5p | 4,2 | 0,0120 | 0,1131 |
|  | miR-1228-3p | -3,1 | 0,0124 | 0,1146 |
|  | miR-92a-1-5p | 3,8 | 0,0131 | 0,1185 |
|  | miR-130b-5p | 2,2 | 0,0133 | 0,1185 |
|  | miR-625-3p | 3,8 | 0,0135 | 0,1185 |
|  | miR-5001-3p | -2,7 | 0,0142 | 0,1209 |
|  | miR-873-5p | -3,4 | 0,0146 | 0,1209 |
|  | miR-8072 | -3,3 | 0,0150 | 0,1225 |
|  | miR-642a-5p | -3,1 | 0,0155 | 0,1229 |
|  | miR-6842-3p | 2,8 | 0,0183 | 0,1343 |
|  | miR-4685-3p | -3,5 | 0,0184 | 0,1343 |
|  | miR-323a-3p | 3,0 | 0,0189 | 0,1343 |
|  | miR-582-5p | 2,7 | 0,0195 | 0,1358 |
|  | miR-4645-3p | 3,8 | 0,0196 | 0,1358 |
|  | miR-18a-3p | -2,2 | 0,0202 | 0,1367 |
|  | miR-654-5p | 3,5 | 0,0206 | 0,1371 |
|  | miR-380-3p | 3,4 | 0,0217 | 0,1419 |
|  | miR-885-3p | -3,5 | 0,0225 | 0,1421 |
|  | miR-200a-3p | -2,4 | 0,0267 | 0,1645 |
|  | miR-429 | -2,3 | 0,0276 | 0,1667 |
|  | miR-550a-3-5p | -2,4 | 0,0288 | 0,1669 |
|  | miR-3614-5p | 3,5 | 0,0288 | 0,1669 |
|  | miR-18b-3p | -3,8 | 0,0289 | 0,1669 |
|  | miR-185-3p | 2,7 | 0,0299 | 0,1708 |
|  | miR-489-3p | -3,8 | 0,0307 | 0,1736 |
|  | miR-382-5p | 3,3 | 0,0310 | 0,1736 |
|  | miR-760 | 2,5 | 0,0339 | 0,1798 |
|  | miR-3200-5p | -3,0 | 0,0345 | 0,1806 |
|  | miR-4738-3p | 3,4 | 0,0362 | 0,1867 |
|  | miR-671-5p | 3,1 | 0,0363 | 0,1867 |
|  | miR-218-1-3p | 3,1 | 0,0378 | 0,1890 |
| **LNIII_vs_CTL** | miR-150-3p | 3,9 | 1,42E-05 | 0,0079 |
|  | miR-423-5p | 2,6 | 7,39E-05 | 0,0205 |
|  | miR-625-3p | 5,5 | 0,0008 | 0,1533 |
|  | miR-150-3p | 4,5 | 0,0017 | 0,1533 |
|  | miR-642b-5p | 4,5 | 0,0016 | 0,1533 |
|  | miR-197-5p | 3,8 | 0,0015 | 0,1533 |
| **LNII_vs_CTL** | miR-1260b | 3,3 | 0,0001 | 0,0041 |
|  | miR-19a-3p | -3,0 | 0,0001 | 0,0041 |
|  | miR-363-3p | -2,2 | 0,0002 | 0,0044 |
|  | miR-16-5p | -2,2 | 0,0004 | 0,0108 |
|  | miR-144-5p | -2,7 | 0,0004 | 0,0108 |
|  | miR-744-5p | 2,1 | 0,0004 | 0,0108 |
|  | miR-330-3p | 2,8 | 0,0005 | 0,0116 |
|  | let-7c-5p | 2,6 | 0,0007 | 0,0146 |
|  | miR-101a-3p | -2,0 | 0,0008 | 0,0169 |
|  | miR-19b-3p | -2,3 | 0,0008 | 0,0169 |
|  | miR-183-5p | -3,1 | 0,0009 | 0,0169 |
|  | miR-409-3p | 2,3 | 0,0010 | 0,0189 |
|  | miR-125a-3p | 3,9 | 0,0012 | 0,0199 |
|  | miR-4435 | 6,7 | 0,0012 | 0,0199 |
|  | miR-4446-3p | 3,1 | 0,0013 | 0,0199 |
|  | miR-320-3p | 2,0 | 0,0013 | 0,0199 |

*We considered all significant miRNAs with FDR <0.2 as a conservative cutoff to discover potential biomarkers.
